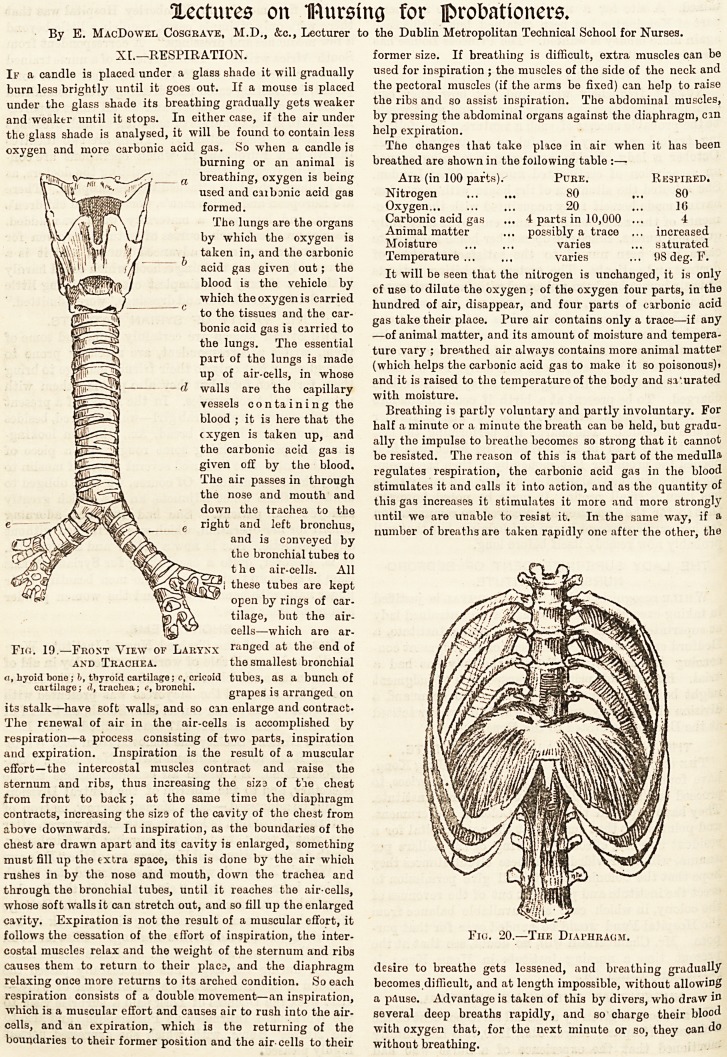# "The Hospital" Nursing Mirror

**Published:** 1900-07-21

**Authors:** 


### "The Hospital" Nursing Mirror.

Being the Nursing Section of "The Hospital."

[Contributions for this Section of "The Hospital" should be addressed to the Editor, The Hospital, 28 & 29, Southampton Street, Strand, London, W.C., and should have the word "Nursing" plainly written in left-hand top corner of the envelope.]

### Notes on News from the Nursing World.

### THE PRINCESS OF WALES AND HER NURSES.

On Friday afternoon the Princess of Wales, after inspecting the "Light Cure" for lupus at the London Hospital went across the road into the nurses' delightful private garden, where an extremely interesting little ceremony was performed. About a hundred nurses had assembled, and after the Hon. Sydney Holland, as chairman of the hospital, had presented the 20 nurses whom the Princess of Wales is sending to South Africa, Her Royal Highness spoke to them in turn. She herself tied round the arm of each the badge of a Princess of Wales' nurse, and then handed to every one of them a Shetland shawl and rug. Each nurse is also taking with her a tin box filled with medical necessities for typhoid fever in as concentrated a form as possible; and the Princess, with characteristic solicitude, is sending by them two large boxes of presents for the soldiers whom they may have to nurse.

### THE FOUNDER OF THE PROPOSED NURSING HOME AT PEKING AMONG THE MURDERED.

One of the victims of the tragedy at Peking, which has horrified the civilised world none the less because it had been prepared for the event, was, there is every reason to fear, the Rev. Gilbert Reid. Our readers will remember that our issue of April 22nd last year contained an article on "A Nursing Home at Peking." Mr. Reid, who was an American missionary, and had spent nearly two decades of his life among the Chinese, was the promoter of an important scheme for establishing an International Institute in China, including a nursing home. The idea of the latter was, of course, to provide the Chinese with some of the advantages which are enjoyed by the sick and suffering in the West. That Mr. Reid should have perished at the hands of the people he was trying to benefit intensifies the regret which will be felt at his sad fate. For the present the entire enterprise he had in view must necessarily fall to the ground, though it may hereafter be the lot of others, in different times, to carry it to a successful issue.

### THE PEKING HOSPITALS.

Although the only ladies mentioned as employed in nursing in Peking were Mrs. Ransome (acting matron), Miss Ransome, and Miss Lambert, as there are four hospitals in the city under English and American control we are afraid that the whole of the staff were among the massacred. Dr. Curwen, who was in charge of the S.P.G. Mission Hospital, was recently ordered home, and his successor has not yet been appointed.

### DR. G. E. MORRISON AS A NURSE.

In the course of an interesting article on Dr. G. E. Morrison, who acted as correspondent of the *Times* in China, and was, according to all appearances, included in the massacre at Peking, our contemporary mentions that he was not content to give merely his advice as a medical man when asked for it. "He used himself to say that in most ailments good nursing can do more than the best doctoring, and he was not the man to preach without giving practical effect to his precepts. Amongst those with whom he met his death at Peking there were not a few whom his skilful and gentle nursing had in happier days helped to bring back to life."

### THE IRISH NURSES OF THE IMPERIAL YEOMANRY HOSPITAL.

No less than half a dozen of the nurses who sailed in the "Dunvegan Castle" for service in the Yeomanry Branch Hospital were supplied by the City of Dublin Nursing Institution. All of them have enjoyed from four to ten years' service. Miss Emily N. Buchanan has been in the employment of the institution for many years, and has been engaged very largely in nursing both at home and abroad. She is a native of county Cavan. Miss Margaret Meade, who comes from county Louth, has also had many years practical experience in the nursing of fever and surgical cases. Miss Charlotte Strahan, who is a native of Dublin, and Miss Ellen Johnson, who comes from Louth, are also highly trained. Miss Flora Fitzmorris is the only member of the contingent who wears the decoration of the Order of Serving Sister of the Hospital of St. John of Jerusalem. It was granted to her for meritorious service in connection with the typhus epidemic among the islanders of Inniskea in 1897. She is a native of County Carlow, Miss Ellen O'Neill, who is a native of Dublin, is also highly qualified.

### THE FOOD SUPPLY TO THE NURSES AT NAAUWPOORT.

Miss Lightfoot, of the Army Nursing Service Reserve, gives an authoritative contradiction to the reports that the food given to the nurses at No. 6 General Hospital, Naauwpoort, is not satisfactory. Miss Lightfoot states that the food has been "remarkably good." It is true, she says, that on the arrival of the nurses it was nearly impossible to get fresh milk, and that vegetables were a difficulty. "But still nearly every day we have potatos and generally one other fresh vegetable, fresh beef and mutton every day, and bacon for breakfast, varied by eggs—these rarely—kidneys and cold ham, jam, and puddings in variety for luncheon and dinner." In fact, Miss Lightfoot confesses that "she never expected to live half so well on active service." Her testimony may be absolutely relied upon, and it reflects favourably on the management of the superintending Sister.

### SISTERS VERSUS ORDERLIES.

"The only thing against a sister," said a private who had been invalided home, wounded at Deelfontein, "is that you mustn't smoke while they're in the room. At least, that's the army orders. And then sisters are more trouble to move about than men. A man's kit weighs about six pounds, and the tighter he packs it the better for his things—they come out with the right creases in them. But you mustn't crush a lady's clothes, and they want about six trunks. But for nursing," he went on, "give me sisters. A man, he says to you, 'Now then, turn over, lift your leg,' but a sister speaks nice, and is like a mother to you. Give me sisters."

### THE GENERAL HOSPITAL, BARBADOES.

Under the heading of "The Unfortunate Hospital," a Barbadoes paper prints the following :—

"We have been informed that Miss Cargill, the head nurse at the General Hospital, has sent in her resignation. There is evidently something radically wrong at this institution, as the head nurses find it impossible to remain there many months. That there should be disorganisation, however, is only what is to be expected at an institution supported by the public, and yet with an administration independent of the public and everyone else."

We are able to throw some additional light on the situation. A lady who was then matron of a hospital in the provinces, was asked early last year if she would care about the post of matron, or head nurse, at the Barbadoes General Hospital; and, liking tropical work, as well as being somewhat tempted by 200 beds more than she had at the time, not to mention what promised to be a much larger salary, she accepted the appointment. She arrived at her destination on May 1st, and after three months' experience she resigned. With the medical and nursing staff she had no fault to find, but she found it impossible to work with the committee. Her successor arrived there about November, and it is obvious that she, too, could not tolerate the position. Now, we hear, printed papers have been sent to some of the hospitals in England inviting applications for the posts of head nurse and assistant head nurses. In the circumstances, and with the warning of the Barbadoes paper before them, nurses holding good appointments at home will certainly do well to think twice before proposing to accept service at "the unfortunate hospital."

### THE NURSES OF GUY'S.

The nursing staff and others of Guy's Hospital have again been entertained by the Treasurer and Mrs. Cosmo Bonsor at Kingswood Warren. The first garden party took place on Monday, July 2nd. The guests, numbering about 130, left London Bridge in a special train for Kingswood Warren, where they were met by Mr. Bonsor, and at once proceeded to the house, either walking or driving. The weather was most unpropitious; notwithstanding the rain, however, the guests seemed to thoroughly enjoy themselves, and after light refreshments were partaken of, some wandered through the rooms, or sat and listened to the Blue Hungarian Band, which was discoursing sweet music in the hall; and others, braving the elements, visited the church, farm, garden, greenhouses, and stables. At five o'clock a cold collation was served in the dining-room, and at half-past six the party took leave of their host and hostess and started for the station. As the train left hearty cheers were given for Mr. and Mrs. Bonsor, and Guy's was reached at a quarter to eight. The second garden party took place on July 9th, the guests numbering about 120. The weather on this occasion was delightful, and while some of the company explored the beautiful woods, others sat under the trees or played croquet or tennis. Refreshments were served in a large marquee in front of the house, and subsequently Mr. and Mrs. Bonsor and family accompanied their guests to the station, many of whom had their arms full of lovely flowers from the garden, and heather and bracken from the moors.

### NURSING AS AN EMPLOYMENT.

It is perhaps hardly to be expected that on a warm July afternoon people will contentedly sit under an iron roof and listen to a lecture, however interesting the subject, on the "Training to Perfection of Children." So it is not surprising that a mere handful of persons were present when Mrs. Ada S. Ballin gave her lecture last week in the Children's Section of the Earl's Court Exhibition; and that Dr. Dutton had not many more in the evening, when he spoke on "Nursing as an Employment for Women." Dr. Dutton maintained that nursing is as much a calling as the priesthood of a Church; that the requirements of a nurse are ability, sound health, an even temper, and skilful training. "There are," he said, "few plums; and a nurse can only expect to get a fair living. A good nurse is the greatest possible help to a physician. There are certainly, out of the nearly 20,000 in the profession, some black sheep, but he hoped that they were getting fewer. With the qualities he had named, and a sincere love of the work, he thought that a girl could not do better than take up the work."

### A RECENT APPOINTMENT BY THE BIRKENHEAD GUARDIANS.

The Medical Officer of Birkenhead Union Infirmary writes us an unnecessarily angry letter. He informs us that the superintendent nurse recently appointed has "the usual three years' certificate" from Chorlton Union Infirmary. In that case there is nothing more to be said. Chorlton is, of course, a recognised training school; and, as we stated last week, the Birkenhead Guardians cannot be blamed for any appointment that fulfils the requirements of the general order of the Local Government Board.

### NURSING FOR THE MIDDLE CLASSES.

In the fourteenth annual report of the Peterborough District Nursing Association it is stated that "occasionally the nurse has been asked by the doctors to attend those who are better off, and would not wish to benefit by a charitable institution, although they are hardly in a position to obtain the services of a trained nurse." It is added by Miss Saunders, the lady superintendent, that in the year she has received from these grateful patients and their relatives the sum of £12 8s. 6d., given in voluntary donations to the funds of the association, in various sums from 5s. to a guinea. We do not, of course, know how far such donations correspond to the means of the patients referred to, but it looks as if a satisfactory step had been taken in Peterborough towards the solution of the problem of nursing for the middle classes where means are strictly limited.

### THE KENSINGTON NURSING ASSOCIATION.

It is unsatisfactory to find that, while the work for the nurses in Kensington increases year by year, the subscriptions from individuals in the court suburb fall off. As it is pointed out in the report which was adopted at the annual meeting, the present staff of a superintendent and six nurses, whose cost is from £850 to £900 a-year, must be diminished if the income is not maintained. A site for a nursing home in the northern part of Kensington has been obtained, but in this case again more money is wanted. The Princess Louise has already contributed £35.

### NURSING ENTERPRISE.

Nurses frequently write and ask how they can establish themselves as visiting nurses, and what would be the probable success of such a venture. The following typical case may be useful as an example. Last October a lady of wide experience in nursing opened an association of certificated nurses in West London. She selected the situation of the house with care, after having made herself fully acquainted with the requirements of the neighbourhood, and employed only fully qualified nurses. Since she opened her home 107 private cases have been nursed to the satisfaction alike of doctors and patients. The system of visiting patients at their homes has been welcomed with favour wherever it has become known, but there has not been sufficient demand for such a nurse's services as to set apart one especially for the work. The fees charged for visits are as follows: 2s. 6d. for a single visit, or 10s. 6d. (sometimes 15s.) a week for one visit daily. For a cancer case which requires dressing twice a day 30s. a week is charged. To be present at a birth if occurring before the nurse engaged arrives 10s. 6d., or 21s. if further service is needed. The charge for performing the last office to the dead is 21s. When the home was first opened the promoter lost money by it, then the incomings and outgoings balanced each other, and lately there has been a small profit. It must be noted, however, that so far, the organiser has received no remuneration for her services except board and lodging, but that will probably now remedy itself before long.

### THE LADY SUPERINTENDENT OF BEDFORD NURSING INSTITUTE.

While recognising that The Hospital is justified in taking exception to the choice of an untrained lady as superintendent of the Bedford Nursing Institute, a Bedford critic begs for "a suspension of judgment concerning the appointment" until the lady has had a trial. But on this principle a suspension of judgment might be asked if a civilian were sent to command a division of the army, or a man who had never practised at the Bar were elevated to the Bench.

### THE HONG KONG NURSING INSTITUTE.

The committee of the Victoria Hospital, Hong Kong, have found it impossible, owing to the rise in prices, to proceed with the building of the Nursing Institute. They have reported this to the Colonial Government, and pointed out that quarters at the new hospital for a resident surgeon, effecting a saving of 720 dollars per annum, will be provided. In these circumstances they hope that the Colonial Society will give permission to erect the institute and pay for it out of the revenues of the colony, in which case any available balance from the Hospital Fund would be handed over for that purpose. Mr. Chamberlain will, no doubt, see that at the present time a nursing institute in Hong Kong is particularly called for.

### NURSING AT BULAWAYO HOSPITAL.

In an account given by a special correspondent of the Bulawayo Hospital, March 31st, 1900, it is incidentally mentioned that the experience of a nurse who had worked five months in Kimberley Hospital was that there were only "simple cases, requiring little beyond a few medicines and dressings." A correspondent from South Africa writes: "The experience of a nurse trained there for three and a-half years was that when competent, interesting cases and dressings were committed to the charge of the nurses, and at all times opportunities were given to nurses of assisting at such dressings and cases." She continues: "There are two theatres (native and European) in which operations are constantly performed, the nurses assisting the doctors, as no medical school is attached to the hospital. There are European and native men's, women's, and children's wards, and last year a maternity ward was added. Besides practical work courses of lectures are given for both junior and more advanced nurses, and it is a thoroughly efficient training school, which could hardly be the case if only the 'simplest cases, requiring little beyond a few medicines and dressings,' were admitted."

### THE VANITY OF SYRIAN PATIENTS.

The Syrian women are certainly vain, and some of them, writes a correspondent, are specially prone to smuggling. "Not only do their friends manage to bring them forbidden food, but they also provide them with numerous toilet accessories. In the locker of a present patient—a very pretty Arab girl—we discovered, besides sundry oranges, bits of bread, &c., a broken looking-glass, a paper containing some rouge, and a piece of French chalk. She had used several pieces of muslin to apply these cosmetics. Of course, we were obliged to confiscate these toilet adjuncts, an act which greatly annoyed the patient. She had intended adorning herself for her operation, which was performed without any anæsthetic. She is now a sadder and a paler girl. It is, however, quite a usual thing for Syrians of this class to make up their faces. The men beautify their finger nails with henna stains, and the women powder and paint their faces."

### SHORT ITEMS.

The nurses of the Trained Nurses' Institute, Weymouth, had a small sale of work the other day in aid of the wounded and sick soldiers and sailors. The Mayor opened the sale, and the Mayoress was presented with a bouquet by the senior nurse, Miss Smith, on behalf of the staff. The sum of £17 was realised.—The East London Nursing Society has removed from 168, Whitechapel Road to 43, Rutland Street, New Road, Commercial Road, E., where all communications to the Secretary, and to the Matron of the Central Division, should be addressed.—Miss Marian Barrow, matron of Savernake Hospital, is leaving, with much regret, after five years' work, to take up nursing in Kimberley, South Africa.—Miss Cecil Hanbury, one of the "Princess of Wales's Nurses," who has just started for South Africa, informs us that "since she left the London Hospital she has for two periods nursed at the Cottage Hospital, Brixham, besides doing district work in her own village and some private nursing."—Miss A. T. E. Mitchell, who was a pupil of St. Michael's Home, Kimberley, during the siege, has successfully passed the London Obstetrical Society's examination of July.—Last week the Duchess of York paid a surprise visit to the Workhouse Infirmary at Portsmouth, and also to the Nurses' Home, with which she expressed herself highly pleased.

### Lectures on Nursing for Probationers.

By E. MacDowel Cosgrave, M.D., &c., Lecturer to the Dublin Metropolitan Technical School for Nurses.

### XI.—RESPIRATION.

If a candle is placed under a glass shade it will gradually burn less brightly until it goes out. If a mouse is placed under the glass shade its breathing gradually gets weaker and weaker until it stops. In either case, if the air under the glass shade is analysed, it will be found to contain less oxygen and more carbonic acid gas. So when a candle is burning or an animal is breathing, oxygen is being used and carbonic acid gas formed.

Fig. 19.—Front View of Larynx and Trachea.

*a*, hyoid bone; *b*, thyroid cartilage; *c*, cricoid cartilage; *d*, trachea; *e*, bronchi.

The lungs are the organs by which the oxygen is taken in, and the carbonic acid gas given out; the blood is the vehicle by which the oxygen is carried to the tissues and the carbonic acid gas is carried to the lungs. The essential part of the lungs is made up of air-cells, in whose walls are the capillary vessels containing the blood; it is here that the oxygen is taken up, and the carbonic acid gas is given off by the blood. The air passes in through the nose and mouth and down the trachea to the right and left bronchus, and is conveyed by the bronchial tubes to the air-cells. All these tubes are kept open by rings of cartilage, but the air-cells—which are arranged at the end of the smallest bronchial tubes, as a bunch of grapes is arranged on its stalk—have soft walls, and so can enlarge and contract. The renewal of air in the air-cells is accomplished by respiration—a process consisting of two parts, inspiration and expiration. Inspiration is the result of a muscular effort—the intercostal muscles contract and raise the sternum and ribs, thus increasing the size of the chest from front to back; at the same time the diaphragm contracts, increasing the size of the cavity of the chest from above downwards. In inspiration, as the boundaries of the chest are drawn apart and its cavity is enlarged, something must fill up the extra space, this is done by the air which rushes in by the nose and mouth, down the trachea and through the bronchial tubes, until it reaches the air-cells, whose soft walls it can stretch out, and so fill up the enlarged cavity. Expiration is not the result of a muscular effort, it follows the cessation of the effort of inspiration, the intercostal muscles relax and the weight of the sternum and ribs causes them to return to their place, and the diaphragm relaxing once more returns to its arched condition. So each respiration consists of a double movement—an inspiration, which is a muscular effort and causes air to rush into the air-cells, and an expiration, which is the returning of the boundaries to their former position and the air-cells to their former size. If breathing is difficult, extra muscles can be used for inspiration; the muscles of the side of the neck and the pectoral muscles (if the arms be fixed) can help to raise the ribs and so assist inspiration. The abdominal muscles, by pressing the abdominal organs against the diaphragm, can help expiration.

The changes that take place in air when it has been breathed are shown in the following table:—

| Air (in 100 parts). | Pure. | Respired. |
|---|---|---|
| Nitrogen ... ... | 80 ... | 80 |
| Oxygen... ... ... | 20 ... | 16 |
| Carbonic acid gas ... | 4 parts in 10,000 ... | 4 |
| Animal matter ... | possibly a trace ... | increased |
| Moisture ... ... | varies ... | saturated |
| Temperature ... ... | varies ... | 98 deg. F. |

It will be seen that the nitrogen is unchanged, it is only of use to dilute the oxygen; of the oxygen four parts, in the hundred of air, disappear, and four parts of carbonic acid gas take their place. Pure air contains only a trace—if any—of animal matter, and its amount of moisture and temperature vary; breathed air always contains more animal matter (which helps the carbonic acid gas to make it so poisonous), and it is raised to the temperature of the body and saturated with moisture.

Breathing is partly voluntary and partly involuntary. For half a minute or a minute the breath can be held, but gradually the impulse to breathe becomes so strong that it cannot be resisted. The reason of this is that part of the medulla regulates respiration, the carbonic acid gas in the blood stimulates it and calls it into action, and as the quantity of this gas increases it stimulates it more and more strongly until we are unable to resist it. In the same way, if a number of breaths are taken rapidly one after the other, the

Fig. 20.—The Diaphragm.

desire to breathe gets lessened, and breathing gradually becomes difficult, and at length impossible, without allowing a pause. Advantage is taken of this by divers, who draw in several deep breaths rapidly, and so charge their blood with oxygen that, for the next minute or so, they can do without breathing.

### Nursing at the Siege of Mafeking.

### A CHAT WITH SISTER GAMBLE.

By a Special Correspondent.

It was rather difficult to secure a chat with Sister Gamble, who was in Mafeking during the siege, but I found her at last, and was glad to see she was not alone in the huge hotel off the Strand, where the only way of finding your friends is to send a page-boy over the building to chant the number of your friend's room. When we had been talking a few minutes two other ladies joined us—Sister Wilson, from Maritzburg, and Sister Flower, from Durban.

"But we hide our heads when Sister Gamble is here," said Sister Wilson, laughingly; "we are nowhere when a siege nurse is present."

But this was denied by Sister Gamble, and we agreed that each had done her best in the circumstances in which she had been placed. Only, when a nurse has been through a siege, and such a siege as Mafeking, she is, of course, an object of special interest.

### In Mafeking Hospital.

"Had you many cases of wounds?" I asked Sister Gamble.

"A great many," she answered. "The Mauser bullet wounds were very bad, but not so bad as the expanding bullets. The expanding bullets tear away the tissues, and are a long time healing. One man who was in hospital with a wound was wounded a second time by a bullet from outside; we had put him in a safe place, as we thought, but a shot struck him. We carried many of the men into the passages, anywhere where we thought they would be safe; and we had often to pick the bullets out of the walls over the beds. The hospital stands high, you see. Once a shell burst in the ward, but only one person was killed, a native boy."

"You had some enteric cases, too, I suppose?"

### Only a Spoonful of Porridge.

"Yes, during the latter months of the siege, and malaria and dysentery too. It was terrible to have nothing to give our patients; sometimes I thought I must run away when dinner time came, and there was only a spoonful of porridge for some of the men, and nothing for the others. And think of having to give a person recovering from enteric a polony made of horse or donkey! The supplies came just before I left, but I did not see them unpacked. I left by the first train after the line was re-opened, and have been invalided home."

"Were you in charge of the hospital!" I asked.

"No; there was a matron, Miss Hill, but as she was ill part of the time I shared the work with her."

"Is it a large hospital?"

"No; it has only thirty beds, but we had to add to them. It is really a civilian hospital, and Miss Hill, Mrs. Parmanter, and I were on the staff. When it was turned into a military hospital we stayed on and worked under the Red Cross Society."

"Had you no one to help you except the orderlies?"

### Outside Help.

"Yes, we had several ladies from the town;" and here the other two sisters joined in, saying how hard the many ladies who really had given splendid help in the hospitals felt it that they were included in the statements made by Mr. Treves.

"But," I protested, "I am sure they were not meant. The ladies Mr. Treves so objected to were ——"

"Society butterflies?" they cried.

"Exactly."

Some of the ladies, it appeared, had taken it for granted that they were meant, and had half decided to give up at once, though all three sisters assured me the work they were doing was excellent.

"At Mafeking," Sister Gamble went on, "we had Mrs. McCallum, who was a trained nurse, but a volunteer in our hospital; Miss Cramer, who was nursing a private case in the town, and who joined us; and Mrs. Stewart, who was not trained at all and knew nothing whatever about a nurse's work; she worked very hard, and I should like her to be specially mentioned. Then there were the Sisters of Mercy at the Roman Catholic Convent," Sister Gamble continued. "For six months they lived in the 'dug-out' (the bomb-proof trenches round the town), and gave up their beds to the soldiers. They were flooded out twice by the rains. And Lady Sarah Wilson also gave very valuable help, taking charge, with some other ladies, of the convalescent hospital, to which our patients were removed as soon as possible."

### A Good Word for the Orderlies.

"It's not always the people who do the hardest work who get the most praise," Sister Wilson remarked. "Now I should be very glad if you would say something about the orderlies. They do work so hard, and many of them have been ill themselves; it's nothing but hard work all day, and more kicks than half-pence! I was in charge of an enteric ward at Maritzburg, and the orderlies were simply splendid, often sick with the smells, and with a great deal to do besides ward work—all the fetching and carrying for patients and nurses. It was useless letting any of the patients help, however willing they might be, for it only resulted in relapses."

### "For England, Sir."

Sister Wilson said there were among the patients one or wo malingerers, tired of the war, and anxious to get home One of these, being asked by the doctor, "Well, my man what are you here for?" replied with excessive candour "For England, Sir!" His honesty won the doctor's heart, and his name was not struck off the list of the "invalided home."

### A Plucky Sister.

"Some people have said," I remarked, "that to a certain extent the soldiers are to blame for the terrible amount of enteric."

"It's not true," Sister Wilson rejoined emphatically. "You may be as careful as you like, and yet you get it. I boiled every drop of water both for my patients and myself, yet I have had enteric twice."

Sister Wilson won the good opinion of the War Office authorities in the Cape by going out from Newcastle (Northumberland) at her own expense, being, as she said, too old for the reserve. She was at once given the charge of a ward in Maritzburg Hospital, and commended for her pluck. The ladies who helped in Maritzburg Hospital, not only by assisting in the wards in many ways, but by giving up their milk and eggs for the patients, were Mrs. Johnson, the wife of the principal medical officer, and Miss Whiteman, who worked very hard at the college. The other sisters in charge were Sisters Russell and Hutton Potts. Sister Russell has volunteered for China.

### "Like Skeletons."

There was a little friendly contention between the sisters as to the rival merits of English and Colonial trained nurses, and then Sister Flower told us how her sister, one of the three who had remained in the Johannesburg Hospital when it was taken over by the Dutch, now bitterly repented her choice, and was anxious for the first opportunity to leave. She told us, too, how, in Durban, she had seen many of the men arrive and go on board for England. "It was a dreadful sight," she said; "they were like skeletons with the skin drawn tightly over them, poor fellows."

But the 750 on board the "Assaye," in which all three had come home, arrived safely; there were relapses, but happily no deaths during the voyage.

### The U.S.A. Hospital Ship "Relief."

BY A NURSE IN THE FAR EAST.

THE following account by a nurse now in China of a visit to the United States hospital ship "Relief" will be of special interest at the present moment:—

My first trip to the "Relief" was made in Chinese waters by means of a sampan, a native boat which provides not only the means of livelihood, but the veritable home and dwelling-place of the family, consisting, perhaps, of three or four generations. How and where the numerous members bestow themselves in the very limited space allotted below the deck remains a mystery—a mystery which one has less desire to unravel when one encounters the mixed odour of fish, smoke, and onions that stand between it and solution.

My boat only revealed five persons—the father, who sat stolidly steering; the mother, who rowed with one hand and fed the baby with the other; and two older children, a boy of six, who took his mother's oar when she dived below, and a girl of ten, who alternately sculled and manipulated the sail. With such frail help, the journey seemed somewhat perilous, but we reached the object of our visit in safety, and our fears were soon merged in admiration of the ship that towered above us. Painted white and green, with the intense blue of the sea and sky as a background she bore out her name indeed.

### THE INTERIOR OF THE VESSEL.

The interior looked cool in white and gold. The floors were stained and slightly polished, and the whole place preserved that spotlessness which seems the exclusive prerogative of Jack Tar. The ship, previously a passenger one, had been converted into hospital use for the soldiers engaged in the Phillipine war; but climatic influences seem to have played more havoc than the projectiles of the enemy, for the majority of diagnoses ran upon the tropical trio, dysentery, fever, and "liver."

There was accommodation for 270 patients—more under stress of circumstance—and the long rows of white iron beds, with their chairs and wool mattresses, soft pillows and bright-coloured rugs, looked comfortable in the extreme. One objection occurred to my mind in considering the above diseases, namely, that of the beds being duplicated one above the other, although of course space was thus gained.

Attached to each was a covered tin receptacle for use either as a spittoon or in case of mal-de-mer. Each ward had its own full-sized bath—fitted with hot and cold water and shower spray—its ice box, electric fans and filters, the latter made in such a way that percolation resulted in an ice-cold drink from the tap.

The main kitchen, bright with its copper fittings, was situated conveniently for all patients sufficiently convalesced to fetch the separate trays upon which their diets were served, while further on all sorts of dainties in the shape of jellies, ice-cream, and special diets emerged from a smaller room where a skilful nurse reigned supreme. The cooking here was all done by electricity, and the same force rotated an electric fan, which lessened the burden of her toils.

### APPLIANCES UP TO DATE.

The operating theatre was a gem, and contained every modern appliance. The instruments, hanging in an air-tight case, were polished to a degree that defies description, and this is saying much when one takes into consideration the combined inroads of tropical humidity and sea air.

Different coloured glass bowls were used for the lotions, and the bottles, in addition to being held in place by holes cut to fit, were safeguarded from concussion in rough weather by means of an adjustable bar. Sterilisers, hot-water apparatus, with thermometer to register the temperature, and a huge indiarubber affair much upon the principle of a wind-sail, for irrigation purposes, were also to the fore. Dainty little packages containing sterilised iodoform or other gauze in about one-yard lengths, recommended themselves for ambulance work upon the field, and occupied but very small space. Bandages of varying widths were also treated in this way, and, sealed in their separate packets, appealed to the aseptic heart. Every bullet wound is subjected before operation to the scrutiny of the Röntgen rays, the apparatus for this being the largest I have seen either at home or abroad.

### A LAUNDRY DEPARTMENT.

The dispensary, testing-room, and other offices were fitted in a way that suggested unlimited money, and more than the average common sense. The entire supply of soda water, ice, and electricity is manufactured on board, the consumption of the former naturally being enormous. There is also a special department for laundry purposes, where the clothing is washed, mangled, dried, and aired by steam, the arrangements all being carried out by men.

A steriliser, resembling the cylinder of a huge engine, was worked from the same department, and absorbed mattresses and such other bedding as was destined for disinfection in a truly wholesale way.

The one exception to the treatment was apparently the coloured rugs, which are always destroyed. This, if sterilisation be worth anything, seemed unnecessary waste, as surely what is sauce for the goose is sauce for the gander.

### AN EMBALMED CORPSE ON BOARD.

Between decks, when exploring the workshops, &c., I came across a huge tin case covered with the Stars and Stripes, in front of which reposed a stack of cabbages which some men were busily employed in preparing for dinner. The flag, although in such a queer place, suggested but one idea, and inquiry proved that the corpse of a man (embalmed three months previously) lay underneath ready for shipment to New York when an occasion offered.

Formalin was the disinfectant used, and this, in strong solution, had been injected into the veins and smeared all over the body, its efficacy being unquestionable—an assurance which eased my mind considerably upon the subject of the cabbages and dinner. The inspection of a well-filled up-to-date library, of a polyphon with its wide range of tunes, and an organ played either manually or mechanically, served to show the interest of Americans in their sick, for they had all been gifts.

### NURSES WITHOUT CAPS.

The nurses, drawn from army sources, were very genial and nice, but lacked smartness owing to their capless condition and the want of uniformity in the dresses, which, while all were white, offered a variety of material and style. Doubtless Manila was partly accountable, as laundry work presents difficulties there, and as yet "clear starchers" are an unknown quantity upon the "Relief." The one pretty stylish thing noticed was a collar shaped deep in the front and fastening behind, which gave somewhat the effect of a clerical appearance and avoided the use of brooches or ribbons, which scarcely ever secure a collar neatly. Their real army dress, consisting of white skirt, blue bodice, and scarlet sash, had been laid aside for the more useful white, and the nurses seemed to appreciate the change.

### THE SLEEPING ACCOMMODATION.

A peep into the nurses' bunks, the tiniest conceivable places in which two were closeted but only one could dress at a time, brought our visit to a close, and we departed with a higher opinion of hospital ships than we had previously thought it possible to entertain. For my own part, however, I have no desire to exchange a comfortable lofty room for the Liliputian chamber of an American army nurse, the one serious blot in the 'Relief's' otherwise perfect arrangements.

### Nursing in Sanitoria for Consumption.

By Jane H. Walker, M.D.Brux , Physician to the New Hospital for Women, and Medical Superintendent of the East Anglian Sanatorium.

### I.

The so-called Open-Air treatment of consumption is now a household word, and very few grown-up people—at any rate very few who are in the habit of reading the newspapers—can have failed to come across more or less frequent references to the subject. For two years past there has been what in commercial circles would be called a "boom" in the treatment of consumption. It began with the meeting at Marlborough House, and the formation of that excellent organisation, the Association for the Prevention of Tuberculosis, and we see the vigour with which the crusade against tuberculosis is being carried on by the numerous sanitoria which are cropping up here and there, by the activity of various public bodies, municipal organisations, County Councils, Local Boards of Health, and so forth, in making and enforcing laws for the better housing of what we call the working classes, in exercising vigilance over the hygiene of food, by more efficient inspection of meat, by subjecting samples of milk and butter to careful analysis, and by carrying out careful experiments upon the question of the dangers arising from the consumption of those articles of food in which the tubercle bacilli may flourish.

### A Word of Caution.

All these and similar efforts can only tend towards the amelioration of the public health, and so indirectly towards stamping out consumption, yet one word of caution is necessary. The liberty of the subject should be curtailed as little as possible. That there is danger of a great deal more interference with people's ways and customs than is in any way desirable is shown by several signs of the times. One instance only will suffice. A medical officer of health in the country a few weeks ago expressed an opinion that a local bye-law should be passed to force parents to boil all milk before giving it to their children, or failing that, that dairies should be compelled to deliver all milk sterilised. Let no one misunderstand me when I say, better a few babies should perish than that the population should be subjected to the dominion of such quasi-scientific doctrines.

### The Sanatorium Treatment.

To return to our more immediate subject, the open-air treatment of consumption—or better, the hygienic treatment—or better still, the sanatorium treatment. This treatment was first carried into effect by Dr. Brehmer, of Görbersdorf, in Silesia. Like many other good things, it was made in Germany. Doctor Brehmer thought that sanatoria must be erected in districts where there was no consumption; in other words, that certain regions of the earth were immune, and that there consumption could not exist. We now know that the tubercle bacillus, which is the immediate cause of consumption according to our present knowledge, can thrive and flourish anywhere. The subject was first brought under my notice eight years ago, and since then I have carried out the prin[illegible]s of the rational treatment of consumption w[illegible] possibility of doing so has arisen. The ration[illegible]l treatment consists in a permanent supply of pure, fresh air, in a plentiful and suitable dietary, in carefully arranged periods of exercise, alternating with regular periods of rest, in avoiding all worry and anxiety, and last, but not least, in careful and constant medical supervision. It must be quite obvious to anyone that such a condition of things can only be fully realised in a community, and hence the formation of sanatoria for consumptives. It must also be obvious that if pure air be an essential in the curative treatment of consumption, hospitals for the diseases of the chest which are in the middle of towns can do little beyond the merest palliation. An ideal sanatorium must be far removed from other dwellings, and must be sheltered from winds by being placed on a slope.

### The Nursing.

The nursing required in a sanatorium differs according as to whether the establishment is for well-to-do paying patients or for the poor. It is distinctly a new departure for a nurse to have the charge of better-class patients for such a consider able time as is usual in a sanatorium. Of course, in private nursing and in a nursing home it is only the well-to-do who are being nursed, but there the rule is generally that the same nurse should not take a case for more than three months consecutively; in a sanatorium patients stay from six months to a year or more. This necessarily brings its own difficulties. Many nurses would object to the monotony of the work after the constant variety of cases they may have been accustomed to in hospital work, or the less varied but still fairly frequent change of cases experienced in private nursing. Then, too, as a sanatorium for consumptives must be at some distance from a town or thickly-populated centre, the nurse's life is necessarily very isolated, and she is thrown for society entirely upon the inhabitants of the sanatorium. This will be referred to later on when the question of nurses hours of work and off-duty are considered.

### Nurses who Dread Draughts.

The "open-air" element, especially in the winter, would undoubtedly deter many nurses. Nurses too often belong to that aggravating class of persons which expresses itself as being fond of fresh air but not liking a draught. My heart always sinks when I am brought into contact with such persons, for I know by experience that wherever we are they will wish to have every cranny hermetically sealed, and one is divided between a desire to be polite, and to promote one's own and other people's welfare. It is a neat point to decide on the best method of behaviour under such trying circumstances. However, for the nurse who is afraid of a draught there is comfort of a substantial kind, for she gets used to it in two or three days, and very soon she becomes in her turn a terror to the fresh-air-but-no-draught people, for she finds it next to impossible to sit in any room for more than a very short time with the windows shut. This is not a fad, as her friends, more in sorrow than in anger, call it, but a cultivated necessity of her nature. Any nurse unaccustomed to a really fresh airy house will undoubtedly suffer very considerably if she embarks on her duties at a sanatorium in the winter, but she will become acclimatised, and that very quickly. She may, of course, be allowed to wrap up as much as she likes under her dress, but she should not do her work huddled up in a cloak or shawl, or shiver and look miserable, and whatever discomfort she is feeling she should show no sign of it to her patients.

### The Duties of a Nurse.

What are the duties of a nurse at a sanatorium? Alas! to a nurse whose ideal of work is the accident ward of a London hospital they will seem tame indeed. A few years ago there was a craze for going to Norway to learn the Sloyd system of wood carving. A friend of mine who was bitten with this idea was one of the earliest pupils at Naes, and on her return I asked her to describe the system. "Oh!" she said, "it is mainly whittling." Now that, to my mind, describes nurses' duties at a sanatorium. Little that is exciting, not often a sudden and great rush of work, often possibly no real nursing as an ordinary hospital nurse understands it, and yet it is a nurse's work and not a housemaid's. A new nurse coming to me lately said to the head nurse, "But this is only housemaid's work; I am passionately fond of nursing, but this is not nursing." This nurse came to me later on to discuss her work, and by that time she saw—as she expressed it—that "even dusting can be done so as to make it desperately interesting."

"Who sweeps a room as for Thy laws
Makes that and the action fine."

### A Book and its Story.

### MARIE CORELLI'S NEW STUDY FOR PARENTS.

OPINION may differ as to the literary value of Miss Corelli's latest book compared with earlier ones, but, as to its interest, its sound sense—not always a characteristic of this gifted authoress—its pathos, and the painful realism of two characters at least, there can be no doubt. She calls her present story a "sketch," and the character of "Boy,"* who should be the hero, but who, through hereditary taint, inherited from an inebriate father, fails, from lack of moral-stamina and strength of will, to fulfil the promise foreshadowed by the sweet child-picture given of him, is one outlined with no uncertain indications, showing clearly the certain trend of this hapless victim of an undisciplined childhood, and neglected youth. "Boy," like its predecessor, "The Mighty Atom," is a study for parents to ponder over and lay to heart. Unfortunately, books written with a "purpose" are too often passed over by those to whom their moral specially applies.

An extract from the opening chapter will suffice to show something of the environment in which the tiny "Boy," otherwise Robert D'Arcy Muir, found himself: "From his feeding-chair he saw strange sights—sights which often puzzled him and caused him to beat monotonous time on his plate with his bâton spoon in order to distract his little brain. Two large looming figures occupied his horizon—'Muzzy' and 'Poo Sing.' 'Muzzy' was the easy-going, stout lady with the felt slippers, who gave him bread and milk and said he was her boy. 'Poo Sing' was, in the few tranquil moments of his existence, understood to be 'Dads,' or 'Papa.' 'Boy,' somehow, could never call him either 'Dads' or 'Papa' when he was seized by his staggering fits; such terms were not sufficiently compassionate for an unfortunate gentleman who was subject to a malady which would not allow him to keep steady on his feet without clutching at the mantel-piece. . . . 'Boy,' lately arrived from the Infinite, was guiltless of his present dubious surroundings. He did not make his 'honourable' father a drunkard or his mother a sloven. He came into the world, perchance, to be the redemption of both of his parents, had they received his innocent presence in that spirit. But they did not."

To turn from sordid, repulsive details of the domestic ménage of the D'Arcy-Muirs to a description of that most charming personality, Miss Letitia Leslie, is a refreshing change: "Miss Letty had many faithful friends and advisers, but none more devoted, with the instinctive, intuitive first love of a child, than 'Boy.' He, with wondering eyes, was given to compare 'Muzzy,' whose hair suggested the woolly stuffing of an arm-chair, with the radiant vision known to himself as 'Kiss-Letty' enshrined in his sad little heart as an angel of light, to be dwelt upon in thought, at any rate, when the perplexities of his small life became unsolvable. Miss Letitia Leslie—a wonderful vision to Boy's admiring eyes—a rustling, glistening dream, made up of dove-coloured silk and violet-scented old lace, and tender, calm blue eyes, and small hands with big diamonds flashing on their dainty whiteness. Miss Letty, as she was generally called; 'that purse-proud old maid,' as Captain the Honourable, Boy's father, frequently designated her. Around 'Miss Letty' all the charm of a sweet old-world atmosphere lingers. Her strength and womanliness, her unselfishness, her life, whose sun went down with the death of a lover, lived since in, and for others, is a quite charming picture of a gentlewoman of means and leisure; who, lovely in youth, was lovely and graceful in middle age."

* "Boy." By Marie Corelli. (Hutchinson and Co., Publishers. Price 6s.)

Not less delightful in his way is her friend, Major Desmond, warm-hearted and irascible, who at forty-five was a bachelor still for sweet Letty's sake. Fortunately, she had a saving sense of humour, and the scenes in which the two appear are among the brightest and most amusing in the book. He is impatient, and irritated that his beloved lady should waste the best years of her life in single unblessedness because of her fidelity to, as he knew, an unfaithful lover. "The Major had a secret in his soul which, had he declared it, would well nigh have killed Letitia Leslie; he knew that the man she had loved, and whose memory she honoured with such faithful devotion, had been nothing but a heartless scamp, who in an unguarded moment had avowed that he was going to throw over Letty for "a much prettier and wealthier woman"; but he had never got back from India to carry out his intention, and Letty believed that he died loving her and her only. Who would have undeceived her?—not Dick Desmond, certainly." "And 'Miss Letty's' faith in her worthless lover was so perfect that she was content with her loneliness, knowing that it was only for a little while—that soon she and her beloved would meet again never to part." Miss Corelli, we know, finds much interest in the Unseen, and in questions which with our present limited knowledge of psychic force must remain open. As an instance of the over-mastering power of prayer for those beloved and gone, apart from personal merit, she remarks, "It would be foolish to deny the probability of noble thought radiating to unmeasured distances and affecting for good those who are gone, whom we loved on earth and whose present state and form of life we are not as yet permitted to behold. Anyway . . . whatever wonders be hidden behind Death's dark curtain, it may be conceded that the unfaithful soul of the man was in no wise injured by Miss Letty's prayers, but rather strengthened and sustained." Then the girl of the story is as nice as her name, Violet. She is Major Desmond's niece, and, next to Miss Letty, holds the first place in his heart. Miss Letty has her especial *protégé* in "Boy," and between her dreams for his future and her interest in Violet, who comes to share her home, all her benevolent schemes are divided. Violet, like Miss Letty, has the gift of a faithful heart, and suffers in consequence in devotion to an ideal which fails her. Her uncle's opinion of his sex, formed from an intimate observation of the scamps who invariably attract the best women, had little patience with his niece for breaking her heart over a man who had forsaken her for another man's wife, and then, when she deserted him, came back to Violet for forgiveness and reinstalment. Devotion to a true soul, his own to Miss Letty for instance, through years of unsuccessful pleading, he could understand; but devotion to a worthless reprobate, of whose unworthiness Violet was aware, was what he could not permit. "I shall never care for anyone else," said Violet to her uncle. "Nonsense!" said the Major; "this is your first love, and one gets over first love like the measles." "Did you?" asked Violet anxiously. "God bless my soul! Of course I did. When I was nineteen I fell in love with my father's cook. She was a very pretty woman, and made jam puffs divinely. But she married the grocer, and I lived through it. I was nearly thirty when I found Letty, and I have loved her ever since. Don't worry yourself, thin yourself, or lose your looks. Nobody will thank you except your female friends," which was a sage and quite true suggestion. Violet becomes a hospital nurse. Had Miss Letty "loved only what were worthy of her love," she would have been a happier woman; but then her story would not have been written, and readers would have lost a charming and sympathetic presentment of a very lovely character.

### The Principles of Antiseptic Surgery and the Preparation of Patients for Operation.

Abstract of a Lecture to Nurses given at the City Orthopædic Hospital, June 12th, 1900, by J. Jackson Clarke, M.B.Lond., F.R.C.S., Surgeon to Out-patients at the North-West London and City Orthopædic Hospitals.

I do not hesitate to ask your attention to this subject, trite as at first sight it may appear. The lesson of Listerism cannot be too often read and pondered. Cases of suppuration in wounds made by the surgeon, though now, happily, most rare, do still occur in conditions in which it might appear that every precaution had been taken against infection. In one such case that occurred under my own observation, an exhaustive examination into the mode of preparation of the patient's skin, the boiling of the instruments, the solution of carbolic acid in which they were placed at the operation, the lint swabs, the sutures and ligatures, and finally the dressing, revealed no flaw in the series of precautions. We found, however, that one of the nurses had a slight suppuration at the edge of one of her nails, and I ha e no doubt that in this lay the source of mischief. After a long spell of hospital work, residents in hospitals—whether nurses, house surgeons, or dressers—become run down in health, their resisting powers against the ubiquitous germs of suppuration are diminished, and septic foci, whether in the shape of sore fingers, sore throats, or, if they have any decayed teeth, gumboils, are apt to arise and to be a source of danger. Anyone, whether nurse or surgeon, who has a septic throat or finger requires a holiday, and should at any rate be excluded from the scene of an operation as rigorously as if they had scarlet fever or diphtheria. All surgeons and nurses should be careful to have their teeth seen frequently by the dentist. There is another source of infection that may, unless carefully guarded against, defy our best efforts. I refer to suppurative foci in the patients themselves. This is most frequently observed after circumcision and other operations where the wound cannot be absolutely shut off from the chance of infection. Such cases turn up at the hospital a few weeks after operation without any healing having taken place, but instead free suppuration from an unhealthy granulating surface, which is surrounded by an angry swollen skin, and the glands in the groins are also swollen. In one of these cases the glands remained enlarged, and I was obliged to remove them; they were typically tuberculous, and contained the specific bacilli. What is the cause of these failures in cases of circumcision? They are, in my opinion, nearly always cases of auto-infection from pustules, or scabby patches (impetigo) present on the face, fingers, legs, or other parts of the patient's skin before the operation, and the wound becomes infected from these surfaces. Before a patient is submitted to operation such suppurating foci should be got rid of, and at the same time the diminished resisting-power of the patient, which has led to their being established, should be improved. Head-lice are very frequently the inoculating agents in these conditions, and they must be utterly destroyed or completely removed, if necessary, by shaving the head, before any operation is done. These points I have dwelt upon at some length because I am deeply impressed with their importance. If they are not duly observed all our many details of aseptic and antiseptic surgery may be in vain; the success of operations, and even the lives of patients, will be imperilled, and in hospital work we have to remember that we are the stewards of a generous public, and we are responsible for the perfect carrying out of the work they enable us to undertake.

### The Preparation of the Patient.

Remove all visible hairs by carefully shaving the skin. This should never be left to be done in the operating-room. In operations about the face, head, and genitals, and amputations of the limbs, the shaving is a most important element in treatment.

### The Preparation of the Skin.

The first thing to be done is to remove all dirt and fatty (sebaceous) secretion from the skin. Ordinary soaps do not remove the fat, and in order to loosen this it is necessary to use either turpentine or an ether soap. The latter I find to be less irritating to the skin and more easily washed off than turpentine. A simple and useful formula for an ether and spirit soap has been devised by Mr. Peach, the dispenser of the North-West London Hospital. It is as follows: Soft soap, 2lbs.; spirit (meth. unmineralised), 1 pt.; macerate 24 hours and decant; equal parts of above and ether meth. Sp. g. ·720. After the skin has been freely treated with this soap, which should be well rubbed and kneaded upon the skin in order to remove the secretion in the orifices of the pilo-sebaceous follicles, the soap should be completely washed off by ample rinsing with sterilised water. It has been found that the water from a hot-water apparatus that is in good working order is sterile as far as pathogenic organisms are concerned. The water should be received into vessels that have been wiped out with a strong antiseptic solution, say, 1-20 carbolic.

A few woids now on antiseptics. Some years ago, when I was in charge of a bacteriological laboratory, I made a pretty long series of experiments on various antiseptics. I found that a 1-5,000 solution of biniodide of mercury and potassium killed anthrax spores on silk threads in one minute, whereas a solution of mercury perchloride of the same strength had no effect in the time. My results with carbolic solutions were more favourable to this antiseptic than those of some other observers have been. I found that 1-40 carbolic destroyed anthrax spores on threads in one minute when *no fatty substances were present*. If a dressing of lint wet with 1-40 carbolic lotion be placed on the skin of the region to be operated on for one hour before the operation, after thorough use of ether soap, the part will be sufficiently prepared.

### Resignation.

Kensington District Nursing Association.—Miss Elizabeth Brooke, superintendent of the Kensington Home, resigned her appointment last Tuesday. Having been trained at Middlesex Hospital, Miss Brooke, in 1886, passed on to the Central Home, Bloomsbury Square, for a further course of training in district nursing under Miss Mansel (now Mrs. Cheadle), and from there was sent to Kensington as one of the nurses on the staff when that association was being first started. For nine and a-half years Miss Brooke worked in the Kensington district as a nurse, gradually attaining seniority, and for four years and three months she filled the office of superintendent. Now, after nearly fourteen years in Kensington, she gives up the work, not to lead an idle life, but has offered herself to the Women's Missionary Association in connection with the S.P.G. to work as one of their missionaries abroad. Before leaving, the committee presented Miss Brooke with a cheque for £21 and a handsomely bound teachers' prayer-book. The nurses on the staff presented an escritoire; the patients in the district gave her a handsome gold curb brooch; one old patient a morocco leather purse; some of the old nurses separate useful gifts as tokens of kindly remembrance; and other friends connected with the Home various presents for household purposes, and books. Miss Brooke hopes to sail for India in October.

### Echoes from the Outside World.

### AN OPEN LETTER TO A HOSPITAL NURSE.

The worst has happened in China. All the more favourable reports which allowed us at least to hope a few days back were, it appears, invented by the Chinese so that they could lead up to the dreadful massacre which they knew we must hear of sooner or later. As far as can be gathered at present, not a single man, woman, or child has escaped from Pekin to tell the tale, but from Chinese sources—principally Sheng Taotai, of Shanghai—it is not difficult to learn the gruesome details. With the characteristic cruelty of their race, when they found the Legations were so well defended that they could not finish their work at a stroke, the Chinese poisoned the water supply, and a fearful picture rises before one's eyes of the little children dying in pain, whilst their mothers knew hardly whether to be thankful or to pray that they might be spared. A letter written a few days before the end says that the Legation ladies "were A1, just as plucky as possible," and that the previous night the writer had lent his revolver to a lady, using a Martini rifle himself. Then, one evening at six o'clock the Chinese artillery fire opened upon the British Legation, and though a breach in the wall was soon made, the fighting of the Europeans was so desperate that the besiegers were kept at bay, and Prince Ching and his men came to the assistance of those in the Legation. But in vain; the Boxers were too numerous. One old general of seventy, Wang Weng Shao, led his troops in person, and died fighting to help us, and Prince Ching fell seriously wounded. Thus all night the struggle went on, till as the sun rose fully about seven the next morning the little remaining band of Europeans met death stubbornly, fighting hand to hand to the last. Two men, one fearfully wounded in the head, stole out of the gates of the city, but they, too, were captured and butchered.

Great anxiety is now felt about the position in Shanghai, and the probability of a disturbance in Canton opens up further alarming prospects. Fortunately, the latest intelligence from Tientsin is satisfactory, though the allied troops suffered heavy losses in their successful engagement with the natives. The only pleasant news from afar is that Colonel Wilcocks has redeemed his promise and relieved Kumasi. But for more stirring events the brilliance of this achievement would have received fuller recognition.

Now that the weather is really quite summerlike the Queen must be glad of the refreshing sea breezes at Osborne. Her last appearance in London before she left was the occasion of a great ovation. I was at Hyde Park Corner shortly before Her Majesty arrived from Paddington, and the approaches to the Palace were as densely crowded as if the populace of London had had no chance of seeing their Sovereign for years past. She looked wonderfully well as she drove by, and so did Princess Beatrice, the latter in deep mourning still. She was accompanied by her daughter in pale blue, such a nice frank-looking girl, still sufficiently unaccustomed to State functions to look interested in all that was going on around her. Did you read of the Queen's kind message to Thomas Scott, the engine driver on the North-Eastern Railway, who was run over by an engine at Newcastle Station, where he had endeavoured to rescue a collie dog which had strayed on to the line? Unfortunately both his legs had to be amputated, and in forwarding a cheque Sir Fleetwood Edwards, whilst alluding to the fact that the Queen never allows her name to appear in a subscription of a similar nature, added that Her Majesty hoped the enclosed £10 might "be of some temporary assistance to Mrs. Scott under the circumstances in which he is placed." She also commanded the writer "to express her admiration for the act of humanity of Thomas Scott," and asked "that the expression of her sincere sympathy should be conveyed to the poor fellow."

Nurses in the Australian colonies will learn with interest that the Earl of Hopetoun is to be the first Governor-General of the Australian Commonwealth. Lord Hopetoun, who relinquishes the much-coveted post of Lord Chamberlain of the Royal Household in order to represent the Queen again at the Antipodes, was very popular as Governor of Victoria, and there is reason to believe that the choice was specially desired by the Australian delegates. Although not yet forty, he has been successively Lord-in-Waiting to Her Majesty, Lord High Commissioner to the General Assembly of the Church of Scotland, Governor of Victoria, Paymaster-General, and Lord Chamberlain. In each capacity he has shown remarkable tact, and earned the praise of all sorts of people. His salary is to be £10,000 a year, and he is so wealthy and generous that he will be sure to entertain on a scale of great liberality. Lady Hopetoun is a charming hostess, and the second son, who was born in Australia, is named Charles Melbourne.

I wonder what is your opinion about carnivals? The charity for which the procession is organised must, perforce, benefit a little, and a good many folks, especially the little ones, like to see the siht. There the merits of the case seem to end. The scum of the earth apparently look upon carnivals as their own special fête days, for not only are enormous sums collected by those whose motto is "Charity begins at home," and who stick to all they can get, but thieves secure purses, watches, and scarf pins galore, either with or without violence, whilst tradesmen lose large sums in having to suspend business early so as to avoid damage to their premises. In addition, a sufficient amount of money is spent on the decoration of cars and the dressing of houses along the route to help half a dozen widows of Reservists to bring up their children decently, or to keep a bed in a hospital going for several years. Decidedly these festive shows are not in high favour with the magistrates. They produce too much work at the courts next day; and, as Mr. Denman remarked whilst trying numerous cases on the morrow of one of them, "These carnivals seem to be got up chiefly for the benefit of pickpockets."

It cannot be too widely known that marriage with foreigners is sometimes attended by very deplorable consequences to the bride. Thousands of women in England are probably not yet aware that a ceremony which is perfectly valid here may not be recognised on the other side of the Channel. A very sad instance of this state of affairs was mentioned the other day by the British Chaplain in Paris. An English widow residing in Paris accepted a suitor for her hand, and at his desire agreed to be married in England. The wedding accordingly took place in a London church last May, and the bride having then told the British chaplain of the event, he found that the necessary steps had not been taken to legalise it. The man, on being questioned, cynically informed his wife that he knew perfectly well that the marriage was not legal in France, and thought that she did. In order to frustrate the designs of scoundrels, no Englishwoman should consent to marry a Frenchman in England, unless he produces a certificate signed by the French Consul to the effect that there is no impediment to the ceremony in France. The refusal of French parents constitutes a barrier, and their consent is, in fact, essential, whatever may be the age of the person contracting the marriage. The stipulation seems ridiculous, does it not? But since it is the law of the country, Englishwomen with a weakness for fascinating Frenchmen ought to be on their guard against the dece[illegible]tion which it encourages.

### Everybody's Opinion.

[Correspondence on all subjects is invited, but we cannot in any way be responsible for the opinions expressed by our correspondents. No communication can be entertained if the name and address of the correspondent is not given, as a guarantee of good faith but not necessarily for publication, or unless one side of the paper only is written on.]

### UNIFORM.

"Nurse T." writes: I quite agree that something should be done as regards untrained women wearing uniform. It would be nice if a badge or medal could be worn outside the uniform to certify that the owner is a fully-trained nurse, the badge or medal only to be obtained by showing the original certificate and paying a fee. Medals could be of white metal or silver.

"Ida" writes: I am pleased to see suggestion by "M. S." which is, in substance, my own, long ago. Discard it altogether. What valid reason is there for a nurse wearing a distinctive outdoor costume more than a medical man? Aping sisterhoods and ostentatiously gratifying conceit and vanity! And who could compel them against their own wishes to parade the abominable, inconvenient, and unsuitable full-blown bonnet and fall, and circular, fish-woman cloak? No one! What should we say or think of a medical man advertising himself by some *outre* costume?

"A Nurse" writes: May I, through your columns, say a few words respecting the wearing of nurses' uniform? It appears to be nowadays not the nurses' uniform, but the uniform of anyone who feels inclined to wear it, and sometimes by persons who do no credit to the nursing profession. For that reason many of those who have given much time and work, and have been trained for nurses, are debarred from wearing it. Could not some rule be made by which only those who are really nurses should be entitled to use the uniform, and thus save many nurses the extra expenses of providing themselves with private outdoor clothes?

### A COMPLIMENT TO NURSE.

"Outsider" writes: In Kensington the other day I had occasion to pass the end of Bedford Gardens. Coming towards me were two young women of the acknowledged coster type, low "bangs" over their forehead, hats smothered in uncurled feathers and battered flowers, and unmended frocks. Suddenly I saw the face of one light up, and seizing the arm of her friend, she pointed to the home of the Kensington District Nursing Association. "There," she said, "that's where that dear old nurse lives." The expression of the girl's face was remarkably involuntary testimony to the work the nurses are doing in Kensington.

### BUXTON DISTRICT NURSING ASSOCIATION.

The Hon. Secretary writes: A paragraph in the number of The Hospital "Nursing Mirror" of January 27th date having recently come before the notice of the Buxton District Nursing Association, the committee are anxious to correct the statement that "the financial support given to the association was not what it ought to be in a town of such importance as Buxton." The wants of the association have always been most liberally responded to, and when an appeal was made at the annual meeting for an extra £50, it was subscribed.

*** It will be observed that the inference is to a note which appeared in our columns nearly five months since. We are glad that the extra £50 asked for at the meeting to which we referred has been subscribed.—Ed. *T. H.*

### A SEASONABLE SUGGESTION.

"District Nurse" writes: "Guy's Nurse" will be glad to know that several of the railway companies have decided to allow every first-class passenger 150 lb., every second-class 120 lb., and every third-class 100 lb. free luggage, although their Parliamentary powers enable them to make a charge for excess above 120 lb. first-class, 100 lb. second-class, and 60 lb. third-class. While on the subject of fares, and since the Queen's Nurses have been so recently discussed, may I say what a large and serious item the fares of the London Queen's District Nurses are to the training homes? The nurses have bags to carry, and often find distances so great that unless they ride they cannot cope with the amount of work to be done. Would not the London County Council help them—unless they are already doing so—in some way, either by season tickets or passes?

### THE COLONIAL NURSES IN SOUTH AFRICA.

"An Englishwoman" writes: It seems to me very shameful that we nurses who have been working in South Africa, for the most part English trained, who have in addition the experience of South African diseases, should be spoken of as Colonial nurses, who were employed because there were not enough sent out from England. By Colonial, I mean South African, Canada, and Australia sent nurses. Apparently, South African nurses are only good enough to be shelled by the Boers and undergo the hardships and privations of siege and war. The Dutch turn us out of Johannesburg because we are not fit to touch them, being Englishwomen. The English refuse to employ us, because we are not good enough to nurse English soldiers either. Are English people losing their common-sense? Their treatment of the Anglo-African nurse looks very much like it. Even if not English trained, the South African nurse has a thorough knowledge of enteric, dysentery, and malaria, which is more than can be said of the English nurse, who has never been abroad. A long residence in Africa makes the former much more suited to the country. The nurses on the register of the Colonial Medical Council are trained sufficiently to pass a fairly stiff examination. Had the military authorities employed only those who were registered, there would have been no foundation for the complaint that untrained nurses were working in these hospitals.

### ROMAN CATHOLIC NURSES.

"A Catholic and an Englishwoman" writes: As a member of the Catholic Nurses' Association and the matron of a hospital for women I beg to reply to the letter from the "Matron of a Maternity Charity" in your issue of July 7th. Perhaps she is a member of a Church protected, endowed, and supported by the State, and hardly realises the demands on the charity and the terrible anxieties to meet those demands, which the Roman Catholic Church in this country experience; but more particularly I would draw attention to the great difficulties we Catholics have in England in obtaining posts in hospitals and charities which we are expected, and in many cases bound, to support. In my own experience I, and many others I know, have been repeatedly rejected as candidates for posts on account of our religion in spite of holding the best qualifications, testimonials, and experience obtainable in Great Britain. Our religion has been an objection in institutions which accepted Agnostics, Theosophists, and other persons holding similar doctrines for posts in which the holders were responsible for the welfare of the living and the dying. In the face of this can those who criticise us so harshly expect a great increase of material support from our already over-taxed resources or generous offerings from those who have no privileges and scarcely toleration?

### BOSTON CITY HOSPITAL, U.S.A.

"A Graduate of Boston City Hospital" writes: When I was nursing in America I was often beset by misstatements about English institutions. I see, however, that the habit of criticising without troubling about facts is not confined to the other side of the Atlantic. The author of the papers on "Nursing in America" says, speaking of the Boston City Hospital and the Massachusetts General: "Everything, especially as regarded all pertaining to surgery, is very perfect in both hospitals, but what struck me most was how on earth the nurses managed to get an all round training. Everthing is so specialised. One hospital has no children's ward, the other a very small one. Neither hospital has either an obstetric or an eye ward. Diseases of the ear and throat go elsewhere. The City Hospital has an infectious diseases block, and the other hospital none. Yet these hospitals, like all others, turn out their nurses full graduates, able to nurse any and every case." An American reading this would infer that English training schools always turn out their nurses able to nurse any and every case—that the average English nurse with a three-year certificate from a general hospital has been taught to nurse ophthalmic, aural, infectious, children's diseases, and maternity cases. Is this true? Personally I have not found it so. Of course, in Boston as in London there are special hospitals for special diseases, but there, as here, a considerable number of cases come to the general hospitals. 1. The City Hospital has both aural and ophthalmic services with special operating rooms. The cases are nursed in various rooms set apart. Four are always reserved in Ward R for ophthalmic cases. Many of the aural cases are nursed in the general wards. 2. Ward O, the surgical children's department, contains one ward and six rooms. Thirty-five cases can, I believe, be accommodated. The children's medical cases are nursed in different rooms in wards L and A; a few in the general wards. At the south department for infectious diseases, containing over four hundred beds, the majority of the patients are children. 3. The department for women's diseases is a hospital in itself, with a special service of doctors, a theatre, two wards, and eight rooms. It fills one wing, and has over sixty beds. There are no lying-in wards. How many London hospitals have them? Both I and several American friends would feel most grateful if some English nurses would tell us which London hospitals offer more facilities for "an all-round training," especially as the author admits that "everything . . . is very perfect. I should like to add that the Boston special hospitals offer every advantage in their power to nurses taking a "post-graduate course." The City Hospital has made special arrangements at the Boston Lying-in for their graduates to have six months' training, at the end of which time, if they satisfy the examiners, they obtain a certificate. The nurses are paid during their training, both there and at the other hospitals. The great evil in the nursing system in America is that the nurses depend so entirely on the doctors. Naturally, they have to solicit their interest, and equally naturally the doctors consider them pests for so doing. But then we must remember the individual nurses do not advertise themselves in any other way, which is more than can be said of many English nursing institutions. That the average rate of payment is from 25 dols. to 21 dols. per week; that a strange nurse, after having registered only four days, had four cases in rapid succession, is, I think, sufficient proof that the profession is not overcrowded to any extent. The B.C.H. nurse who had never seen a hip-joint disease case should join forces with the St. Thomas's nurse who was frequently quoted to me. She told a doctor who was attending an emergency maternity case, "She had never washed a baby, and felt afraid to try." The results of perfect systems are not always perfect.

### Death in Our Ranks.

We regret to announce the death of Nurse Kathleen Howard, who died at Beddington, Surrey, of pneumonia, at the early age of 29. She was only appointed last month charge nurse to the Isolation Hospital, Wimbledon, and had previously been engaged at the Croydon Borough Hospital, Waddon. She was for some time at the North Devon Infirmary, and before that she was district nurse at Welbeck, Notts. A correspondent says that Miss Howard was highly esteemed by all who knew her.

### Presentations.

Mill Road Infirmary, Liverpool.—Dr. John Hay, who has just resigned his appointment, has been the recipient of a massive silver inkstand from the nursing staff of this institution.

### OUR CONVALESCENT FUND.

We acknowledge with thanks the receipt of a postal note for 7s. 6d. from A. S. C.

### Council Meeting of the Royal British Nurses' Association.

Sometimes virtue is its own reward, especially when the particular virtue in question is punctuality. It was so on Friday last, when the Royal British Nurses' Association held its council meeting at 11, Chandos Street. The chairman, Dr. Gage Brown, opened the proceedings at five o'clock, and by ten minutes past the business had been disposed of, with workmanlike rapidity.

First the secretary, Miss G. A. Leigh, read the notice convening the meeting, and the minutes of the last meeting. These having been confirmed, Dr. Gaulton moved the re-appointment of the honorary officers, who had, he said, safely steered the association into comparative quiet. This was carried unanimously. Then Dr. Schofield proposed the re-election of the Executive Committee, with the following new members: Medical men, A. Fairbank, M.D., F. J, Wethered, M.D., and C. Percival White, M.D. Matron. Miss Katherine Scott. Sisters and nurses, Mrs. Thompson, Mrs. Hind, Miss Lemon, Miss Rumball, Miss Ainsworth, and Miss Wethered. They were elected unanimously. Miss Katherine Scott is matron of the Sussex County Hospital, Brighton.

The Chairman said that a small attendance was taken in the City as a proof of confidence; he hoped it meant in this case that the members of the Council were satisfied with the state of affairs. He remembered the days of sorrow through which the association had passed; there was now peace, and with the hope that there would soon be peace in South Africa he declared the meeting closed.

### Appointments.

St. Saviour's Infirmary, East Dulwich Grove.—Miss E. M. Byles has been appointed First Assistant Matron. She was trained at Addenbrooke's Hospital, Cambridge, and St. Thomas's Hospital, and was subsequently night nurse and then sister for six years in the same institution. She has since been second assistant matron at St. Saviour's Infirmary, East Dulwich Grove.

### Minor Appointments.

Shoreditch Infirmary.—Miss E. S. Clements has been appointed Ward Sister. She was trained at Mile End Infirmary, and has since been ward sister at Poplar and Stepney Infirmary, and Brook Fever Hospital.

Swindon and Highworth Union.—Miss Margaret J. Roulston has been appointed Superintendent Nurse. She was trained at Kent and Canterbury General Hospital and Wolverhampton Hospital, and has since been night superintendent nurse at Greenwich Union Infirmary.

Wakefield Infirmary.—Miss Florence May Birkin has been appointed Sister. She was trained at the Marylebone Infirmary, and has since been at the Northern Fever Hospital and charge nurse at the Union Infirmary, Keighley.

### To Nurses.

We invite contributions from any of our readers, and shall be glad to pay for "Notes on News from the Nursing World," or for articles describing nursing experiences, or dealing with any nursing question from an original point of view. The minimum payment for contributions is 5s., but we welcome interesting contributions of a column, or a page, in length. It may be added that notices of entertainments, presentations, and deaths are not paid for, but, of course, we are always glad to receive them. All rejected manuscripts are returned in due course, and all payments for manuscripts used are made as early as possible at the beginning of each quarter.

### For Reading to the Sick.

"THE Lord shall guide continually, and satisfy thy soul in drought."—*Isa.* lviii. 11.

So tired! I fain would rest;
But, Lord, Thou knowest best.
I wait on Thee.
I will toil from day to day,
Bearing my cross, and only pray
To follow Thee.
So tired! My friends are gone,
And I am left alone,
And days are sad.
Lord Jesus, Thou wilt bear my load
Along this steep and dreary road,
And make me glad.

So tired! Yet I might reach
A flower to cheer and teach
Some sadder heart;
Or for the parched lips perhaps might bring
One cup of water from the spring
Ere I depart.
So tired! Lord, Thou wilt come
To take me to Thy Home,
So long desired.
Only Thy grace and mercy send,
That I may serve Thee to the end,
Though I am tired. —*M. E. Townsend.*

**Reading.**

Abandon yourself to His care and guidance, as a sheep in the care of a shepherd, and trust Him utterly. No matter though you may seem to yourself to be in the very midst of a desert, with nothing green about you, inwardly or outwardly, and you may think you will have to make a long journey before you can reach the green pastures. Our Shepherd will turn that very place where you are into green pastures, for He has power to make the desert rejoice and blossom as a rose.—*H. W. S.*

The Christian suffers reverently as the child of God, and accepts both patiently and intelligently the trials of life as a Father's loving discipline. He recognises the true purpose of the divine discipline—the formation of a holy character in the face of moral evil. He believes that God only gives pain in order to purify, and that He chasteneth us "for our profit, that we may be partakers of His holiness." "And there should be no greater comfort to Christian persons than to be made like unto Christ by suffering patiently adversities, troubles, and sicknesses. For He Himself went not up to joy, but first He suffered pain; He entered not into His glory before He was crucified. So truly our way to eternal joy is to suffer here with Christ."—*V. Staley.*

"When St. Paul says, 'It became Him for whom are all things, and by whom are all things, in bringing many sons unto glory, to make the Captain of our salvation perfect through sufferings,' it is manifest that he is declaring an universal law; that he is revealing a connection which must exist between Christ and all His members; that they are to become perfect in the same way, according to the same principle of life; that they are to be united not merely with His humanity, but with His suffering humanity. . . Each one's sorrow is a wave in that deep sea of sorrows in which God Himself once lay prostrate."—*T. T. Carter.*

### Wants and Workers.

M. FORREST begs to thank all those who so kindly answered her appeal for old uniforms.

### Notes and Queries.

**THE Editor is always willing to answer in this column, without any fee, all reasonable questions, as soon as possible.**

**But the following rules must be carefully observed:—**

**1. Every communication must be accompanied by the name and address of the writer.**

**2. The question must always bear upon nursing, directly or indirectly.**

**If an answer is required by letter a fee of half-a-crown must be enclosed with the note containing the inquiry.**

*Dispensing.*

(155) Lady wishes for the address of the dispensary in the Isle of Wight, where they teach and take pupils.—*L. N. E.*

Apply the Resident Dispenser, Ryde Dispensary, Isle of Wight.

*Paris.*

(156) I should be very grateful if you would kindly give me any information concerning the English hospital in Paris—as to whether it is a desirable place to be in, and the best method of getting on the staff. —*C. T. B.*

Apply to the Matron, the Hertford British Hospital, Rue de Villiers, Levallois-Perret. It is a nice little hospital of 34 beds, under the management of English trained nurses.

*Fractures.*

(157) I should be glad if you could inform me if there is a new treatment for fractures by movement and massage. If so, when and where has the treatment been tried, and if it proved successful.—*Enquirer.*

Massage is sometimes resorted to very shortly after a fracture has been reduced, in order to keep the muscles supple. But it is only done in special cases under the direction of the surgeon in charge of the case It has been attended with very good results.

*Co-operation.*

(158) Could you kindly tell me whether, either at Exeter or Salisbury, there is a co-operative association for private nurses which would take a percentage on their cases and permit them to live at home when disengaged?—*A. H. M.*

The Exeter Trained Nurses' Institute, Colleton House, Exeter, and the Institution for Trained Nurses, Salisbury, are the nursing institutions managed by a committee at the places named. You must write to the lady superintendents for the terms of engagement.

*India.*

(159) Can you tell me if, in India, Ceylon, or any other of our British hot colonies in the East or elsewhere, there are any distinctly "isolation" hospitals for infectious fevers and other diseases of an infectious nature needing isolation? Also, if they exist, will you kindly tell me where and to whom I must apply for further information?—*Staff Nurse.*

Apply to the India Office, Whitehall, S.W., and the Colonial Nursing Association, Imperial Institute, S.W.

*Home.*

(160) Could you tell me if there is any home or institution in the neighbourhood of Birmingham where a working man, suffering from paralysis agitans could be received at a moderate charge?—*A. W.*

It is possible that the Jaffray Branch Hospital, Gravelly Hill, Birmingham, might receive the case, but the patient must pass through the General Hospital first. The admission to other public homes for chronic invalids is hampered with votes and elections. You will find a list of such institutions in "Burdett's Hospitals and Charities."

*L.O.S.*

(161) I shall be most grateful if you can tell me at which of the London infirmaries I can be trained in midwifery, and prepared for the L.O.S. examination? I have written to very many hospitals, but their fees are, alas, more than I can afford, but think it is possible that the training may be had at an infirmary or workhouse.—*E. S.*

The matron at Fulham Union Infirmary, St. Dunstan's Road, Hammersmith, would give you the terms for training in force at that institution. You date your letter from Liverpool, why not train at the Liverpool Ladies' Lying-in Charity? The fees to non-resident pupils are only £7 7s.

*Visiting Nurse.*

(162) 1. Can you tell me if there is any opening in London or any large town for a trained nurse (non-resident) to assist a doctor with patients in his own house? 2. Also I should be very much obliged if any nurse would give me the benefit of her experience of daily nursing in any large town, usual remuneration per hour, constancy of work, or any particulars.—*Sister C.*

1. You can only hear of such openings through friends or by watching advertisements. 2. Visiting nurses for the better-class patients who have a connection with the medical men of the district frequently succeed well. The usual charges are from 2s. 6d. per visit. See "Nursing Enterprise" in this week's "Mirror."

**Standard Books of Reference.**

**"The Nursing Profession: How and Where to Train." 2s. net.**
**"The Nurses' Dictionary of Medical Terms." 2s.**
**"Burdett's Series of Nursing Text-Books." 1s. each.**
**"A Handbook for Nurses." (Illustrated.) 5s.**
**"Nursing: Its Theory and Practice." New Edition. 3s. 6d.**
**"Helps in Sickness and to Health." Fifteenth Thousand. 5s.**

**All these are published by THE SCIENTIFIC PRESS, Ltd., and may be obtained through any bookseller or direct from the publishers, 28 & 29, Southampton Street, London, W.C.**

### Travel Notes.

### LIII.—UP AND DOWN THE RHINE—COLOGNE.

With the exception of an article dealing with Coblenz last year, my readers and I have not travelled together in the delightful country of the Rhine. I know no locality better calculated to suit all tastes. Pedestrians and cyclists may revel in long expeditions, and return with that visible self-complacency so irritating to their less robust friends, whilst these latter unfortunates, assisted by frequent trains, steamers, and rowing boats, may emulate their achievements in a more humble way.

### The Journey and Living Expenses.

If you go by the Netherland steamboats the journey is extraordinarily cheap. First-class return to Cologne £1 14s. 6d., and second return £1 4s., allowing you thirty days between London and your return. The entire distance is covered by boat, and you have nothing at all to do with the rail. The only disadvantage is that the travelling is rather slow (three days from London to Cologne), but if you are not pressed for time it is delightful, so calm and restful after the high pressure at which most of us live now. If you cannot manage this the next cheapest and best way is via Harwich and the Hook, £3 7s. 6d. first-class return, and £2 4s. 9d. second. With a little management and wisdom you can keep your hotel expenses down to five marks (five shillings) per day, and in the smaller places to four marks.

### Cologne and Its Cathedral.

One is so apt to think that what is familiar ground to oneself must be equally so to others that I was about to pass Cologne unnoticed, but no doubt many of my readers have never seen that most wonderful cathedral, or Dom, as it is called, and would like a few details as to what to look for somewhat more condensed than those of the admirable but expensive Baedeker. You must then stop one night in Cologne if you come by boat, but if you make the journey by rail viâ the Hook, you will have three hours to spare, and can snatch a hasty glimpse at the Dom. This is, however, very unsatisfactory, and there is a good deal to be seen in the city besides its far-famed cathedral. I shall hope, therefore, that you will decide to rest one night and enjoy that marvellous building in ease and comfort. You cannot do better, if economy is necessary, than to go to the Rheinischer Hof unter Fettenhennen—it is close to the cathedral and the station; a man can take your luggage on a truck, and so save a carriage.

### Avoid Touts of All Kinds.

The entrance to the Dom is beset with hordes of would-be guides; they are totally unnecessary, shake your head and walk stolidly by them. Cologne is full of them, though Rome and Venice are even still more afflicted with the pest. Between the hours of nine and ten in the morning and three to seven in the afternoon you cannot walk about the Dom on account of Divine service, so you must bear that in mind in making your plans. You have to pay (somewhat exorbitantly) to visit the choir and treasury. A mark and a half for each person is decidedly high, but it would be very grievous to miss seeing the treasury. No building of its size has undergone greater vicissitudes—by the end of the fifteenth century religious enthusiasm had suffered a chill, and all attempt at completing the wonderful structure begun by Gerard in 1248 was abandoned. In 1796 the French used it as a vast hay store, and stole the lead from the roof. Attempts at restoration were made in 1823. The windows are among its greatest glories; those in the north aisle are genuinely ancient, about the year 1508, but those on the south side are modern; nevertheless they are in such admirable taste (chosen by King Lewis of Bavaria) and the colouring is so excellent that they are in perfect harmony with their elder brethren.

### The Treasury and the Three Kings.

The Chapel of the Three Kings no longer contains their venerated bones. After many wanderings, sometimes in honest and reverent hands, sometimes in those absolutely the contrary, they are at last allowed to rest peacefully in a reliquary within the Treasury, where on great occasions may be seen three skulls with their respective names written in jewels across the top. I have not seen them myself, not being there on the proper occasion; but I am told it is so. The reliquary itself is worth a journey to study. The date is supposed to be somewhere about the last ten years of the twelfth century. Besides this reliquary there is a magnificent silver shrine to St. Engelbert, and some monstrances, thickly encrusted with jewels that must be worth untold sums. Baedeker is urgent to send one painfully climbing up to the gallery of the west towers, but I abhor such pilgrimages and detest a bird's eye view of anything. Moreover, it costs one mark, at which my economic soul revolts. I advise you also to resist the sacristan's importunities. These beautiful and majestic creatures are clothed in scarlet and wear hats something like our beefeaters.

### The Eleven Thousand Virgins.

We are most of us acquainted with the story of St. Ursula and her 11,000 virgins, and here we see the church raised in her honour, and where she and her companions are buried. The best and most realistic picture, or series of pictures, on the subject are those wonderful jewel-like representations painted by Hans Memling—when he was ill at Bruges—as kindly payment to the good nuns who had succoured him. You had better take a train to this church—it is only a penny, saves fatigue, and helps to give you an idea of the city. The church of St. Gereod, too, is worth a visit. The interior is very rich and singular in construction, the choir seeming to be in the centre of the church, as one sees sometimes in Spain. You must visit the Rathhaus and St. Martin's church, and by all means go across the river by the Bridge of Boats which leads to Deutz. Also, do not miss the church of Sta. Maria in Capitole. It was built somewhere about 750 by Pepin's wife, who seems to have been a lady of great energy and determination of character. The crypt where she is buried is perfect, and there is some rich carving round the choir. The church stands in a charming little Platz, with a picturesque gateway at the end. In the evening you will take train for Konigswinter, or, if hours admit, take the steamer, which leaves at eight o'clock in the morning. If you are cycling it would be about 20 miles, and an excellent road.

---

### TRAVEL NOTES AND QUERIES.

Rules in Regard to Correspondence for this Section.—A questioners must use a pseudonym for publication, but the communication must also bear the writer's own name and address as well, which will be regarded as confidential. All such communications to be addressed "Travel Editor, 'Nursing Mirror,' 28, Southampton Street, Strand." No charge will be made for inserting and answering questions in the inquiry column, and all will be answered in rotation as space permits. If an answer by letter is required, a stamped and addressed envelope must be enclosed, together with 2s. 6d., which fee will be devoted to the objects of the "Hospital Convalescent Fund." Any inquiries reaching the office after Monday cannot be answered in "The Mirror" of the current week.

The Black Forest (Perseus).—It is still comparatively cheap, but living costs everywhere 6 marks and generally more. 2. The Wehrathal is easily reached from Brennet on the Constance and Basle Railway. You must drive about 36 miles to Todmoostd. The view is, on the whole, better if you drive the contrary way, taking train at Brennet. 3. The Hollenthal can be visited from Freiburg. One of the most beautiful spots in the Schwartzwald is on the rail from Hornberg to Villengen.

Bay of St. Malo (Klopstock).—It has become so popular that cheapness has flown before the invading foreigner. St. Servan is still reasonable and Paramé, living may be had even yet for 6 and 7 francs per day. Dinard is quite out of the question for you, it is as dear as Trouville. If you would like addresses at either of the other places let me know. First-class return to either £2 13s. 6d., second-class £2 1s. 2d.

## Figures and Tables

**Fig. 19. Fig. 20. f1:**